# A Comprehensive Review of Genus *Sanguisorba*: Traditional Uses, Chemical Constituents and Medical Applications

**DOI:** 10.3389/fphar.2021.750165

**Published:** 2021-09-20

**Authors:** Ping Zhou, Jingyan Li, Qi Chen, Long Wang, Jing Yang, Anguo Wu, Nan Jiang, Yuanzhi Liu, Jianping Chen, Wenjun Zou, Jing Zeng, Jianming Wu

**Affiliations:** ^1^ Department of Chinese Materia Medica, School of Pharmacy, Chengdu University of Traditional Chinese Medicine, Chengdu, China; ^2^ School of Pharmacy, Southwest Medical University, Luzhou, China; ^3^ Department of Medical Technology, Faculty of Associated Medical Sciences, Chiang Mai University, Chiang Mai, Thailand; ^4^ Department of Endocrinology and Metabolism, The Affiliated Hospital of Southwest Medical University, Luzhou, China; ^5^ Education Ministry Key Laboratory of Medical Electrophysiology, Sichuan Key Medical Laboratory of New Drug Discovery and Druggability Evaluation, Luzhou Key Laboratory of Activity Screening and Druggability Evaluation for Chinese Materia Medica, Southwest Medical University, Luzhou, China; ^6^ School of Chinese Medicine, The University of Hong Kong, Hong Kong, Hong Kong, SAR China

**Keywords:** genus *Sanguisorba*, *Sanguisorba officinalis* L, *Sanguisorba minor* Scop, chemical constituents, medical applications, traditional uses

## Abstract

Genus *Sanguisorba* (family: Rosaceae) comprises nearly 148 species, distributed widely across the temperate and subtropical regions of the Northern Hemisphere. *Sanguisorba officinalis* L. (*S. officinalis*) has been used as a hemostatic and scald treating medicine in China for a long time. Numerous studies have demonstrated that plant extracts or monomers from *S. officinalis* exhibit several pharmacological effects, such as anti-cancer, anti-virus, anti-inflammation, anti-bacteria, neuroprotective and hepatoprotective effects. The other species of genus *Sanguisorba* are also being studied by researchers worldwide. *Sanguisorba minor* Scop. (*S. minor*), as an edible wild plant, is a common ingredient of the Mediterranean diet, and its young shoots and leaves are often mixed with traditional vegetables and consumed as salad. Reports on genus *Sanguisorba* available in the current literature were collected from Google Scholar, Web of Science, Springer, and PubMed. The Plant List (http://www.theplantlist.org./tpl1.1/search?q=Sanguisorba), International Plant Name Index (https://www.ipni.org/?q=Sanguisorba) and Kew Botanical Garden (http://powo.science.kew.org/) were used for obtaining the scientific names and information on the subspecies and cultivars. In recent years, several *in vivo* and *in vitro* experiments have been conducted to reveal the active components and effective monomers of *S. officinalis* and *S. minor*. To date, more than 270 compounds have been isolated and identified so far from the species belonging to genus *Sanguisorba*. Numerous reports on the chemical constituents, pharmacologic effects, and toxicity of genus *Sanguisorba* are available in the literature. This review provides a comprehensive understanding of the current traditional applications of plants, which are supported by a large number of scientific experiments. Owing to these promising properties, this species is used in the treatment of various diseases, including influenza virus infection, inflammation, Alzheimer’s disease, type 2 diabetes and leukopenia caused by bone marrow suppression. Moreover, the rich contents and biological effects of *S. officinalis* and *S. minor* facilitate these applications in dietary supplements and cosmetics. Therefore, the purpose of this review is to summarize the recent advances in the traditional uses, chemical constituents, pharmacological effects and clinical applications of genus *Sanguisorba*. The present comprehensive review may provide new insights for the future research on genus *Sanguisorba*.

## Introduction

There is a huge reservoir of compounds in nature that might be useful in drug discovery (Chin et al., 2006). According to a report by the World Health Organization, approximately 80% of the world’s population continues to rely on botanical medicine (Ekor, 2014).

**FIGURE 1:**
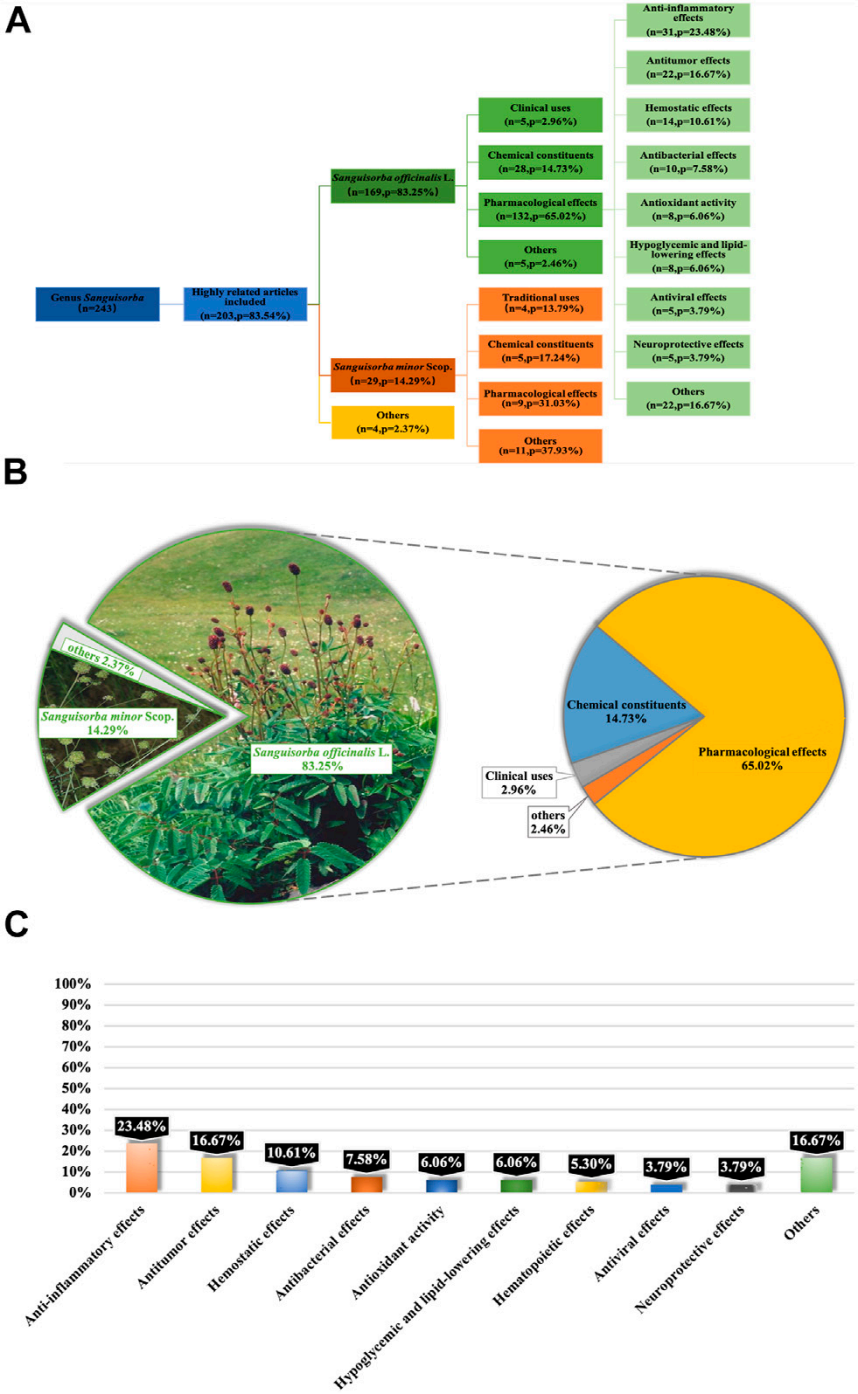
Reports on Genus *Sanguisorba* were Collected from PubMed Database. Systematic classification of retrieved literatures on genus *Sanguisorba* **(A)**; classification and analyzation of the content of the articles **(B)**; classification of reported pharmacological effects of *S. officinalis* **(C)**.

The genus *Sanguisorba* is a member of the Rosaceae family, and the Rosaceae plants have a certain common leaf intergrowth, often accompanied by leaf stipules. The petals are mostly bisexual, and unisexual is relatively rare. The Plant List (The Plant List, 2021), International Plant Name Index (International Plant Name Index, 2021) and Kew Botanical Garden (Kew Botanical Garden, 2021a; Kew Botanical Garden, 2021b) were used for obtaining the scientific names and information on the subspecies and cultivars. Studies have demonstrated that the crude extracts from plants belonging to genus *Sanguisorba* or the purified monomers isolated from the plants of this genus exhibit various pharmacological activities, including anti-inflammatory (Su et al., 2018a; Guo et al., 2019; Yasueda et al., 2020), anti-cancer (Liu et al., 2016; Nam et al., 2017; Tan et al., 2019; Bai et al., 2020a; Liao et al., 2020), anti-lipid peroxidation (Zhang et al., 2012a; Romojaro et al., 2013a; Kim et al., 2018a; Lenzi et al., 2019), anti-bacteria (Su et al., 2019; Zhu et al., 2020a), anti-diabetes (Kuang et al., 2011; Son et al., 2015), hepatoprotective (Stojiljković et al., 2019; Meng et al., 2020), and anti-obesity (Jung et al., 2016; Im et al., 2017; Ji et al., 2018) properties, both *in vitro* and *in vivo* (Figure1).

*S. officinalis,* also referred to as Zi-Yu in South Korea and Japan, Di-Yu in China, and Burnet in the Western nations, has been used as a traditional medicine for a long time (Nguyen et al., 2008). It is used widely in Asia for the treatment of inflammatory and metabolic diseases including diarrhea, chronic intestinal infections, duodenal ulcers, bleeding and diabetes (Zhang et al., 2012b; Seo et al., 2016; Zhang et al., 2018). The pharmacological activity of *Sanguisorba* has been receiving increasing attention from scholars in recent years.

The other species of genus *Sanguisorba* are also being studied by researchers worldwide. One among these species is *S. minor*, which is an edible perennial herb belonging to family Rosaceae, distributed widely across the Sinai Peninsula of Egypt and the temperate regions of Europe, particularly the Mediterranean regions such as Italy (Ceccanti et al., 2019). *S. minor,* as an edible wild plant, is a common ingredient of the Mediterranean diet, and its young shoots and leaves are often mixed with traditional vegetables and consumed as a salad (Romojaro et al., 2013b; Guarrera and Savo, 2016; Karkanis et al., 2019) *S. minor* contains polyphenols in huge amounts and, therefore, has great antioxidant, anti-tumor and antibacterial properties (Ceccanti et al., 2019; Lenzi et al., 2019; Finimundy et al., 2020).

While *S. officinalis* and *S. minor* are the most widespread of all Sanguisorba species, there are also other species in this genus that exhibit different pharmacological effects. So far, over 270 chemical constituents have been identified in the plants of genus *Sanguisorba*, including flavonoids, triterpenoids, steroids, lignans, and organic acids, etc., (Zhao et al., 2017; Jang et al., 2018a).

Reports on genus *Sanguisorba* available in the current literature were collected from Google Scholar, Web of Science and PubMed. The deadline for the literature selected was up to December 2020. We analyzed articles related to not only the species of genus *Sanguisorba* (Figure1A), but also the other two major species of *S. officinalis* and *S. minor* (Figures 1B,C).

The aim of the present report is to review the research advances concerning genus *Sanguisorba,* in terms of its chemical composition, pharmacological activity, toxicology and clinical application, to assist in future drug development and applications involving this species.

## The Botanical Description and Distribution of Genus *Sanguisorba*

**FIGURE 2:**
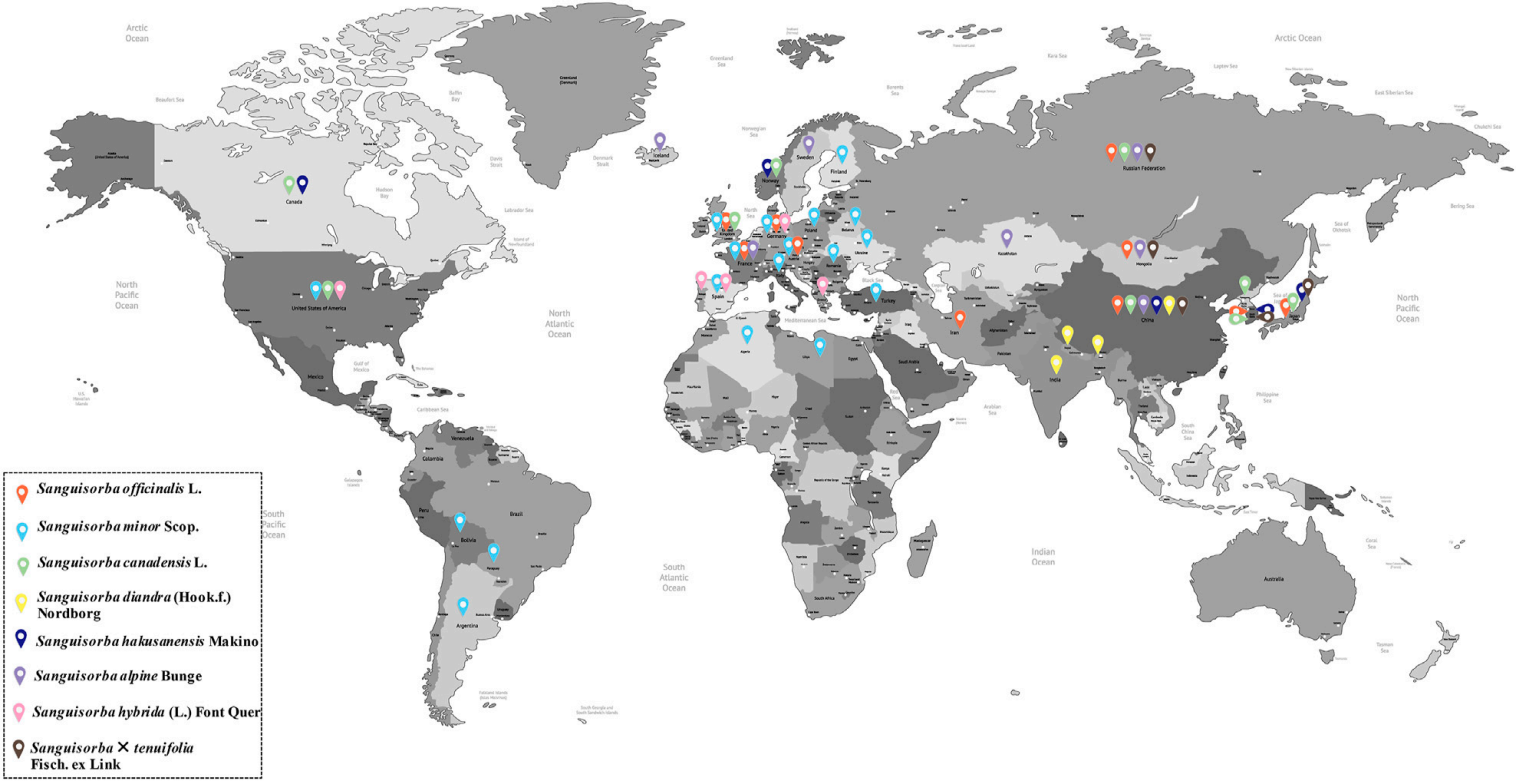
The specific distribution of genus *Sanguisorba.*

Genus *Sanguisorba* comprises perennial flowering herbs belonging to family Rosaceae and includes about 148 species and subspecies distributed mainly across East Asia and southern Europe (Zhu et al., 2019). Figure 2 shows that the specific distribution of genus *Sanguisorba* used for medicinal purposes is cultivated mainly in East Asia, although its cultivation in Europe is also increasing lately. *S. officinalis* and *S. minor* are the most widespread species of genus *Sanguisorba*, and their botanical, biological, and ecological characteristics are discussed in the sections ahead.

### *Sanguisorba officinalis* L.

**FIGURE 3:**
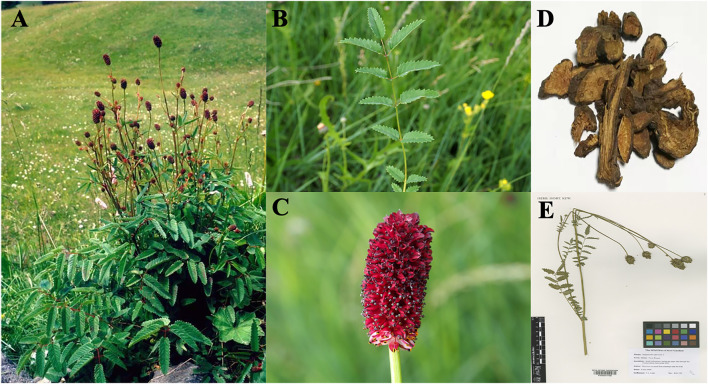
*Sanguisorba officinalis* L. Plants (Kew Botanical Garden, 2021a). The whole plant of *S. officinalis* **(A)**; the leaves of *S. officinalis* **(B)**; the flower of *S. officinalis* **(C)**; Sanguisorbae Radix **(D)**; specimen of *S. officinalis* **(E)**.

*S. officinalis* (Figures 3A–C), which is referred to as Zi-Yu in South Korea and Japan, Di-Yu in China, and Burnet in the western nations, is a perennial plant distributed across a range of geographical regions, including the wet grasslands, hillside meadows, and pastures of the colder regions of Europe and Asia and the northern regions of North America (Bączek, 2015; Zhu et al., 2019). The plants of this species have a height in the range of 30–120 cm and upright stems. Their roots are sturdy and mostly spindle-shaped, while the leaves are pinnate with serrated margins. The flowers are dark red and grow in dense clusters or spikes at the length of 1–7 cm (Bunse et al., 2020). The flowering and fruiting period in this species ranges from July to October mostly (Yang et al., 2015). Pawlaczyk-Graja et al. (2016) believed that the plants of this species exude the scent of cucumber. The seeds of this species have the optimal germination temperature and requirements similar to those of all the other species within genus *Sanguisorba*, although. The seeds of *S. officinalis* germinate more easily than those of the other species under the same conditions, which indicates that this species is better adapted to extreme temperature conditions, which might also be one of the reasons for its wide distribution (Holloway and Matheke, 2003).

*Sanguisorbae* Radix (Figure 3D), which is the dried root of *Sanguisorba officinalis* L. or *Sanguisorba officinalis* var. *longifolia* (Bertol.) T.T.Yu and C.L.Li *(S. longifolia* Bertol*)* according to the documentation in Chinese Pharmacopoeia, has been used as Traditional Chinese Medicine (TCM). The plants are uprooted and collected immediately after germination in spring or after withering in autumn. After washing the plants, the fibrous roots are discarded and the remaining plant is whole-dried or slice-dried for later use in TCM. Similar documentation is available in the Polish Pharmacopoeia, according to which the underground parts of plants (rhizomes and roots) are known for their healing properties (Bączek, 2015).

### *Sanguisorba minor* Scop.

*Sanguisorba minor* Scop. (also referred to as small or salad burnet) is a drought-tolerant species that exhibits extremely high drought resistance (Fry et al., 2018). The plants of this species are approximately 60–105 cm in height and are distributed widely across the Sinai Peninsula, Egypt and temperate regions of Europe (Ceccanti et al., 2019).

**FIGURE 4:**
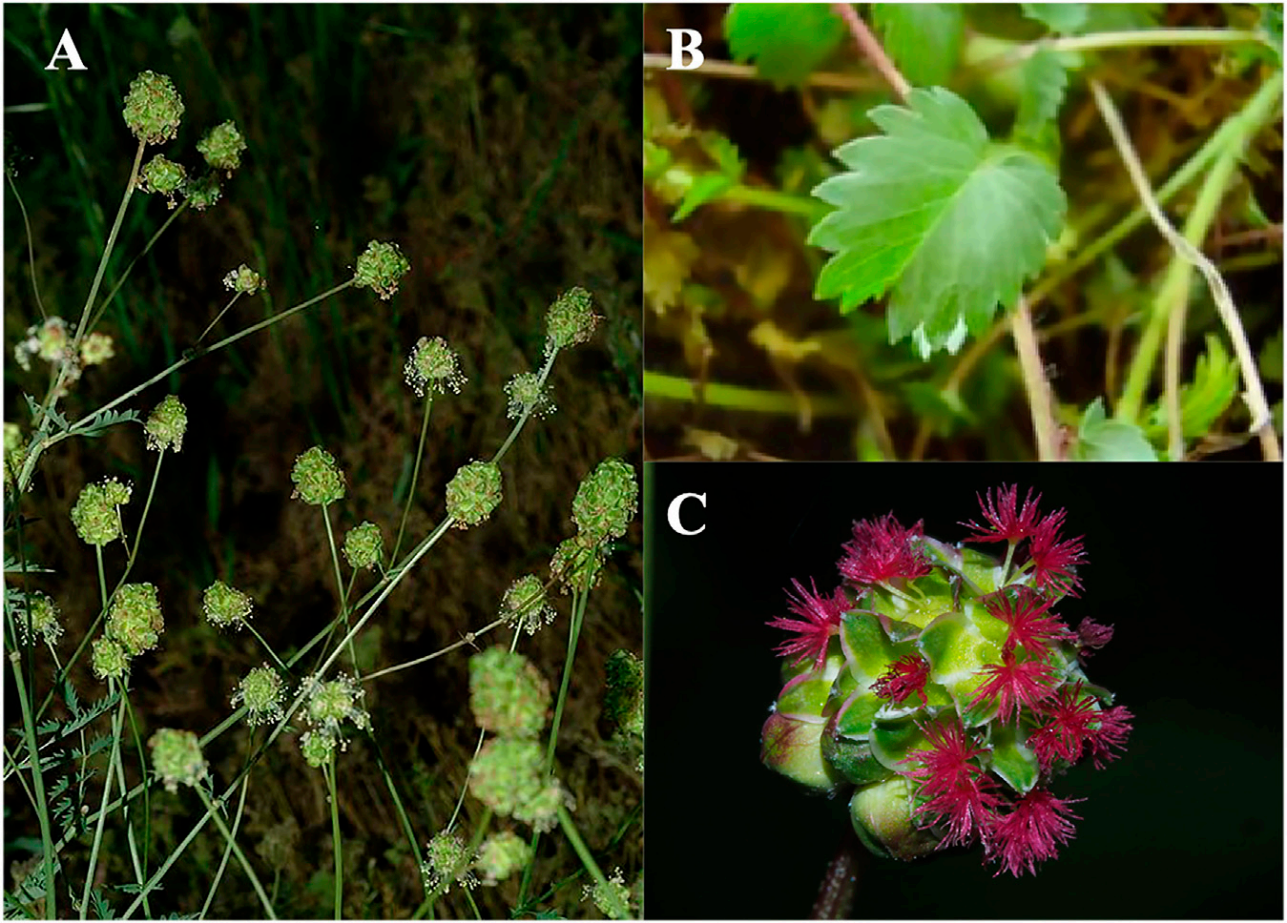
*Sanguisorba minor* Scop. Plants (Kew Botanical Garden, 2021b). The whole plant of *S. minor* **(A)**; the leaves of *S. minor* **(B)**; the flowers of *S. minor* **(C)**.

*S. minor* (Figure 4) comprises edible perennial herbs having pinnate leaves, red-green petals (Cuccioloni et al., 2012), short petioles, and 1–1.5 cm long leaflets growing in pairs or alternately. Inflorescences appear at the end of plant stems. The flowers of this species have four sepals and no petals, with a long peduncle flower head that is globose or ellipsoid and up to 2 cm in length (Paniagua-Zambrana et al., 2020).

## Traditional Uses of Genus *Sanguisorba* Plants

Historical documents state *S. officinalis*, *S. minor* and *Sanguisorba albanica* András. and Jáv. (*S. albanica*) as traditional foods because of their attractive flavor (Caporaso et al., 2015; Lenzi et al., 2019). The plants of these species were added to cheese, butter, ice drink, fresh orange juice, Kiwi juice, and vinegar for flavor enhancement (Sanchez-Bel et al., 2015; Sabbatini et al., 2019). Fresh leaves from young plants are used as a seasoning for salads and meat dishes in certain western nations (Bączek, 2015). In China, *S. officinalis* is regarded as an important tonic food and is frequently added to dishes as such as Diyu porridge, Diyu chitterlings soup, Diyu Huaihua Yin and Shaguo Diyu (Zhao et al., 2017) to diversify the flavors in modern diets.

In addition to being a wild edible plants, *S. officinalis* is also widely used for treating several diseases. *Sanguisorbae* Radix*,* the dry root of *S. officinalis* or *S. longifolia* Bertol*,* has been traditionally used for cooling the blood, clearing heat, healing wounds and alleviating snake bites (Hachiya et al., 2001; Karkanis et al., 2014; Liu et al., 2014; Jang et al., 2018b). In Korea, the whole plant of *S. officinalis* is used slightly differently compared to its roots; while the whole plant is commonly applied for the treatment of diseases in women and bloody stool hemorrhoid pus, the root is mostly used for treating inflammation and for skin regeneration (Kim and Song, 2011). In the Armenian region, the aerial part of *S. officinalis* has been in use as a traditional medicine for treating different diseases (Ginovyan et al., 2020). Studies reported in recent years have provided scientific evidence that the components present in the different parts of *S. officinalis* plant are indeed different (Na et al., 2019). It is reported that the aerial parts of *S. officinalis* contain higher amount of polyphenols compared to its roots (Bunse et al., 2020).

*S. minor* has several similarities with *S. officinalis,* such as both are wild edible species that have also been used in traditional medicine in different regions. Owing to its constituent bioactive compounds, minerals and fiber, *S. minor* is added to omelets and scrambled eggs in the traditional Mediterranean cuisine (Romojaro et al., 2013a). It is also mixed with the common vegetables as a delicacy in Italian cuisine (Guarrera and Savo, 2016).

## Chemical Constituents in the Plants Belonging to Genus *Sanguisorba*

The present report summarizes the research findings reported in the research papers and articles on the chemical component separation and identification of genus *Sanguisorba* plants, published between the years 2008 and 2020 (Table 1), based on, but not limited, to the summary of Zhao et al. (2017), which includes the studies published mainly between the years 2008 and 2014. So far, five species and varieties of this genus have been studied phytochemically, and over 270 compounds have been identified, including flavonoids, triterpenes, phenols, terpenes, fatty acids, and several other types as sterols and neolignans. According to the preliminary pharmacological studies, the extracts and compounds isolated from the plants of this genus exhibit a wide range of biological activities.

**TABLE 1:** Chemical constituents in the genus *Sanguisorba* (2008–2020).

No.	Name	Classification	Plant sources	Extraction solvent andparts	References
**Triterpenes**					
1	Changyediyuine I	triterpenes	*S. longifolia* Bertol	95% EtOH extract of powdered dried roots	Shen et al. (2008)
2	Changyediyuine II	triterpenes	*S. longifolia* Bertol	95% EtOH extract of powdered dried roots	Shen et al. (2008)
3	Changyediyuine III	triterpenes	*S. longifolia* Bertol	95% EtOH extract of powdered dried roots	Shen et al. (2008)
4	arjunic acid	triterpenes (oleanane)	*S. officinal*	ethanol extract of roots	Ponou et al. (2011)
5	2-oxo-3*β*,19*α*-dihydroxyurs-12-en-28-oic-acid 28-*β*-d-glucopyranosyl ester	triterpenes	*S. tenuifolia*	70% EtOH extract of dried roots	Kuang et al. (2011)
6	2*α*,19*α*-dihydroxy-3-oxo-12-ursen-28-oic acid *β*-D-glucopyranosyl ester	triterpenes	*S. tenuifolia*	70% EtOH extract of dried roots	Kuang et al. (2011)
7	2*α*,3*α*,9*α*,24-tetrahydroxyolean-12-en-28-oic acid	triterpenes (ursane)	*S. officinal*	75% ethanol extract of the dried and powdered stems and roots	Wang et al. (2014)
8	1*α*,2*β*,3*β*,19*α*-tretrahydroxyurs-12-en-28-oic acid	triterpenes (ursane)	*S. officinal*	70–80% EtOAc/hexanes extract of the roots	Eyong et al. (2017)
9	3-oxo-15*α*,19*α*-dihydroxyurs-12-en-28-oic acid	triterpenes (ursane)	*S. officinal*	ethanol extract of roots	Wang et al. (2019a)
10	3-oxo-7*β*,19*α*-dihydroxyurs-12-en-28-oic acid	triterpenes (ursane)	*S. officinal*	ethanol extract of roots	Wang et al. (2019a)
11	18,19-seco,1*β*-hydroxyl-3,19-dioxo-urs-11,13 (18)-dien-28-oic acid	triterpenes (ursane)	*S. officinal*	ethanol extract of roots	Wang et al. (2019a)
12	1*β*-hydroxyeuscaphic acid	triterpenes (ursane)	*S. officinal*	ethanol extract of roots	Wang et al. (2019a)
13	19*α*-hydroxy ursolic acid	triterpenes (ursane)	*S. officinal*	ethanol extract of roots	Wang et al. (2019a)
14	ursolic acid	triterpenes (ursane)	*S. officinal*	ethanol extract of roots	Wang et al. (2019a)
15	3-oxo-urs-11,13 (18)-dien-19,28-olide	triterpenes (ursane)	*S. officinal*	ethanol extract of roots	Wang et al. (2019b)
16	(+)-3*β*-hydroxy-ursan-28-oleic acid	triterpenes	*S. officinal*	MeOH/H_2_O and EtOH/H_2_O extract of fresh flowers	Bunse et al. (2020)
17	3*β*-[(*α*-l-arabinopyranosyl) oxy]-urs-12,18 (19)-dien-28-oic acid *β*-D-glucopyranosyl ester	triterpenes	*S. officinal*	MeOH/H_2_O and EtOH/H_2_O extract of fresh flowers	Bunse et al. (2020)
18	2,19*α*-dihydroxy-3-oxours-1,12-dien-28-oic acid 28-O-*β*-D-glucopyranosyl ester	triterpenes (ursane)	*S. officinal*	95% EtOH extract of the dried roots	Wang et al. (2020b)
19	3*α*,19*α*,24-trihydroxyolean-12-en-28-oic acid	triterpenes (oleanane)	*S. officinal*	95% EtOH extract of the dried roots	Wang et al. (2020b)
20	2*α*,3*β*-dihydroxyurs12,18-dien-28-oic acid 28-O-*β*-D-glucopyranosyl ester	triterpenes (ursane)	*S. officinal*	95% EtOH extract of the dried roots	Wang et al. (2020b)
21	7-dimethyl-8-hydroxyoctadien-1-ol	triterpenes	*S. officinal*	95% EtOH extract of the dried roots	Wang et al. (2020b)
**Phenols**					
22	methyl 4-O-*β*-D-glucopyranosy-5-hydroxy-3-methoxylbenzoate	phenols	*S. officinal*	70% EtOH extract of the air-dried roots	Zhang J. et al. (2012)
23	3,3′,4′-tri-O-methylellagic acid	phenols	*S. officinal*	70% EtOH extract of the air-dried roots	Zhang J. et al. (2012)
24	fisetinidol-(4*α*-8)-catechin	phenols	*S. officinal*	70% EtOH extract of the air-dried roots	Zhang J. et al. (2012)
25	*α*–resorcylic	phenolic acids	*S. officinal*	80% methanol extract of air-dried rhizomes and herbaceous tissues	Biernasiuk et al. (2015)
26	*β*–resorcylic	phenolic acids	*S. officinal*	80% methanol extract of air-dried rhizomes and herbaceous tissues	Biernasiuk et al. (2015)
27	protocatechuic	phenolic acids	*S. officinal*	80% methanol extract of air-dried rhizomes and herbaceous tissues	Biernasiuk et al. (2015)
28	gentisie	phenolic acids	*S. officinal*	80% methanol extract of air-dried rhizomes and herbaceous tissues	Biernasiuk et al. (2015)
29	p-hydroxyphenylacetic	phenolic acids	*S. officinal*	80% methanol extract of air-dried rhizomes and herbaceous tissues	Biernasiuk et al. (2015)
30	p-coumaric*(E+Z)*	phenolic acids	*S. officinal*	80% methanol extract of air-dried rhizomes and herbaceous tissues	Biernasiuk et al. (2015)
31	syringic	phenolic acids	*S. officinal*	80% methanol extract of air-dried rhizomes and herbaceous tissues	Biernasiuk et al. (2015)
32	vanillic	phenolic acids	*S. officinal*	80% methanol extract of air-dried rhizomes and herbaceous tissues	Biernasiuk et al. (2015)
33	sinapic*(E+Z)*	phenolic acids	*S. officinal*	80% methanol extract of air-dried rhizomes and herbaceous tissues	Biernasiuk et al. (2015)
34	(-)-epigallocatechin	phenols	*S. officinal*	methanol extract of air-dry raw material	Bączek, (2015)
35	(-)-epicatechin	phenols	*S. officinal*	methanol extract of air-dry raw material	Bączek, (2015)
37	(-)-epigallocatechin gallate	phenols	*S. officinal*	methanol extract of air-dry raw material	Bączek, (2015)
38	(-)-epicatechin gallate	phenols	*S. officinal*	methanol extract of air-dry raw material	Bączek, (2015)
39	astragalin	phenols	*S. officinal*	methanol extract of air-dry raw material	Bączek, (2015)
40	apigenin-O-deoxyhexoside	phenols	*S. minor*	n.d.	Guarrera and Savo, (2016)
41	quercetin-galloyl-hexoside	phenols	*S. minor*	lyophilized young leaves and stems were added to the vegetable oils	Romojaro et al. (2018)
45	taxifolin 3-O-*β*-D-glucopyranoside	phenols	*S. officinal*	MeOH extract of the dried crushed roots	Su et al. (2018a)
46	methyl 3-(*β*-D-glucopyranosyloxy)-4-hydroxy-5-methoxybenzoate.	phenols	*S. officinal*	methanol and Aqueous extract of the roots	Su et al. (2018b)
47	methyl 4-(*β*-D-glucopyranosyloxy)-3-hydroxy-5-methoxybenzoate	phenols	*S. officinal*	MeOH, EtOAc, n-BuOH and water extracts	Su et al. (2019)
48	apigenin derivatives	phenols	*S. minor*	80% methanol extract of aerial parts and roots	Karkanis et al. (2019)
49	chlorogenic	phenols	*S. minor*	80% methanol extract of aerial parts and roots	Karkanis et al. (2019)
50	caffei	phenols	*S. minor*	80% methanol extract of aerial parts and roots	Karkanis et al. (2019)
51	chicoric acid derivatives	phenols	*S. minor*	80% methanol extract of aerial parts and roots	Karkanis et al. (2019)
52	pedunculagin	phenols	*S. minor*	80% methanol extract of aerial parts and roots	Karkanis et al. (2019)
53	B-type*(epi)*catechin tetramer	phenols	*S. minor*	80% methanol extract of aerial parts and roots	Karkanis et al. (2019)
54	sanguiin H-10 isomer 1	phenols	*S. minor*	80% methanol extract of aerial parts and roots	Karkanis et al. (2019)
55	punicalagin gallate	phenols	*S. minor*	80% methanol extract of aerial parts and roots	Karkanis et al. (2019)
56	lambertianin C	phenols	*S. minor*	80% methanol extract of aerial parts and roots	Karkanis et al. (2019)
57	sanguiin H-10 isomer 2	phenols	*S. minor*	80% methanol extract of aerial parts and roots	Karkanis et al. (2019)
58	galloyl-bis-HHDP- glucoside	phenols	*S. minor*	80% methanol extract of aerial parts and roots	Karkanis et al. (2019)
59	ellagic acid hexoside	phenols	*S. obtusa*	80% methanol extract of aerial parts and roots	Karkanis et al. (2019)
60	gallic acid glycoside	phenolic acids	*S* ***.*** *officinal*	70% ethanol extract of dried powder	Zhu et al. (2019)
61	digalloyl hexoside	phenolic acids	*S. officinal*	70% ethanol extract of dried powder	Zhu et al. (2019)
62	brevifolin-carboxylic acid isomers	phenolic acids	*S. officinal*	70% ethanol extract of dried powder	Zhu et al. (2019)
63	trigalloyl-hexoside	phenolic acids	*S. officinal*	70% ethanol extract of dried powder	Zhu et al. (2019)
64	methoxy trihydroxybenzoic acid methyl ester-*O*-sulfate	phenolic acids	*S. officinal*	70% ethanol extract of dried powder	Zhu et al. (2019)
65	ellagic acid-pentose	phenolic acids	*S. officinal*	70% ethanol extract of dried powder	Zhu et al. (2019)
66	ethyl gallate	phenolic acids	*S. officinal*	70% ethanol extract of dried powder	Zhu et al. (2019)
67	galloyl-ellagic acid	phenolic acids	*S. officinal*	70% ethanol extract of dried powder	Zhu et al. (2019)
68	methyl-ellagic acid-pentose	phenolic acids	*S. officinal*	70% ethanol extract of dried powder	Zhu et al. (2019)
69	3,3′-*O*-dimethyl ellagic acid-sulfate	phenolic acids	*S. officinal*	70% ethanol extract of dried powder	Zhu et al. (2019)
70	methoxy trihydroxybenzoic acid methyl ester-*O*-sulfate	phenolic acids	*S. officinal*	70% ethanol extract of dried powder	Zhu et al. (2019)
71	3,4′-*O*-dimethyl ellagic acid	phenolic acids	*S. officinal*	70% ethanol extract of dried powder	Zhu et al. (2019)
72	3,3′,4′-*O*-trimethyl ellagic acid	phenolic acids	*S. officinal*	70% ethanol extract of dried powder	Zhu et al. (2019)
73	cyanidin-galloyl-hexose	phenols	*S. officinal*	MeOH/H_2_O and EtOH/H_2_O extract of fresh flowers	Bunse et al. (2020)
74	cyanidin-malonyl-glucose	phenols	*S. officinal*	MeOH/H_2_O and EtOH/H_2_O extract of fresh flowers	Bunse et al. (2020)
75	*β*-hydroxypro-piovanillone	phenols	*S. officinal*	95% EtOH extract of the dried roots	Wang et al. (2020a)
76	methyl 3-*O*-methyl-gallate	phenols	*S. officinal*	95% EtOH extract of the dried roots	Wang et al. (2020a)
77	chavicol 4-*O*-*α*-L-arabinofuranosyl- (1→6)-*β*-D-glucopyranoside	phenols	*S. officinal*	95% EtOH extract of the dried roots	Wang et al. (2020a)
78	2-di-*O*-galloyl-*β*-D-glucopyranoside	phenols	*S. officinal*	95% EtOH extract of the dried roots	Wang et al. (2020a)
79	sanguisorbaside A	phenolic glycosides	*S. officinal*	95% EtOH extract of the dried roots	Wang et al. (2020a)
80	sanguisorbaside B	phenolic glycosides	*S. officinal*	95% EtOH extract of the dried roots	Wang et al. (2020a)
**Flavonoids**					
81	fisetinidol-(4*α*-8)- catechin	flavonoids	*S. officinal*	70% EtOH extract of the air-dried roots	Zhang S. et al. (2012)
82	quercetin	flavonoids	*S. officinal*	70% ethanol extract of the dried powder	Zhu et al. (2019)
83	epicatechin-(4→8)-gallocat- echin	flavonoids	*S. officinal*	70% ethanol extract of the dried powder	Zhu et al. (2019)
84	taxifolin-7-O-*β*-D- glucopyranoside	flavonoids	*S. officinal*	70% ethanol extract of the dried powder	Zhu et al. (2019)
85	gallocatechin	flavonoids	*S. officinal*	70% ethanol extract of the dried powder	Zhu et al. (2019)
86	isorhamnetin hexoside III	flavonoids	*S. officinal*	70% ethanol extract of the dried powder	Zhu et al. (2019)
87	taxifolin	flavonoids	*S. officinal*	70% ethanol extract of the dried powder	Zhu et al. (2019)
88	isorhamnetin-sulfate	flavonoids	*S. officinal*	70% ethanol extract of the dried powder	Zhu et al. (2019)
89	isorhamnetin	flavonoids	*S. officinal*	70% ethanol extract of the dried powder	Zhu et al. (2019)
90	swertianolin	flavonoids	*S. officinal*	70% ethanol extract of the dried powder	Zhu et al. (2019)
91	baicalin	flavonoids	*S. officinal*	70% ethanol extract of the dried powder	Zhu et al. (2019)
92	okanin	flavonoids	*S. officinal*	70% ethanol extract of the dried powder	Zhu et al. (2019)
93	taxifolin 4′-O-*β*-D-glucopyranoside	flavonoids	*S. officinal*	95% EtOH extract of the dried roots	Wang et al. (2020b)
94	(2R,3R)-(+)-dihydrokaempferol-3-*β*-D-glucopyranoside	flavonoids	*S. officinal*	95% EtOH extract of the dried roots	Wang et al. (2020b)
95	maesopsin-6-O-glucopyranoside	flavonoids	*S. officinal*	95% EtOH extract of the dried roots	Wang et al. (2020b)
96	(−)-gallocatechin	flavonoids	*S. officinal*	95% EtOH extract of the dried roots	Wang et al. (2020b)
97	taxifolin 3-*O*-glucoside	flavonoids	*S. officinal*	95% EtOH extract of the dried roots	Wang et al. (2020b)
**Other compounds**					
98	(7S,8R)-4,9,5′,9′-tetrahydroxy-3,3′-dimethoxy-8-O-4′-neolignan-7-O-*α*-l-rhamnopyranoside	neolignans	*S. officinal*	80% EtOH extract of the roots	Hu et al. (2012)
99	(7S,8R)-4,9,9′-trihydroxy-3,3′,5′-trimethoxy-8-O-4′-neolignan-7-O-*α*-l-rhamnopyranoside	neolignans	*S. officinal*	80% EtOH extract of the roots	Hu et al. (2012)
100	(7S,8R)-4,7,9,9′-tetrahydroxy-3,3′-dimethoxy-8-O-4′- neolignan	neolignans	*S. officinal*	80% EtOH extract of the roots	Hu et al. (2012)
101	9-O-[6-O-acetyl-*β*-d-glucopyranosyl]-4-hydroxycinnamic acid	phenylpropanoid glycosides	*S. officinal*	80% EtOH extract of the roots	Hu et al. (2012)
102	8-O-*β*-d-glucopyranosyl-(R)-(+)-3,4,8-trihydroxy methyl phenylpropionate	phenylpropanoid glycosides	*S. officinal*	80% EtOH extract of the roots	Hu et al. (2012)
103	*β-*sitosterol	sterol	*S. officinal*	80% chloroform-methanol extract of the herb and underground organs	Mirgos et al. (2012)
104	*β*-sitosterol D-glucoside	sterol	*S. officinal*	80% chloroform-methanol extract of the herb and underground organs	Mirgos et al. (2012)
105	campesterol	sterol	*S. officinal*	80% chloroform-methanol extract of the herb and underground organs	Mirgos et al. (2012)
106	stigmasterol	sterol	*S. officinal*	80% chloroform-methanol extract of the herb and underground organs	Mirgos et al. (2012)
107	brassicasterol	sterol	*S. officinal*	80% chloroform-methanol extract of the herb and underground organs	Mirgos et al. (2012)
108	rosamultin	saponins	*S. officinal*	MeOH extract of the dried crushed roots	Su et al. (2018a)
109	kajiichigoside F1	saponins	*S. officinal*	MeOH extract of the dried crushed roots	Su et al. (2018a)
110	(+)-5-methoxyl-cycloolivil	aryl-tetralin-type lignans	*S. officinal*	70% EtOH extract of the whole plants	Wang et al. (2018)
111	(+)-5,5′-dimethoxyl-cycloolivil	aryl-tetralin-type lignans	*S. officinal*	70% EtOH extract of the whole plants	Wang et al. (2018)
112	(2*E*)-7-hydroxy-3,7-dimethyl-2-octenyl 6-O-*α*-L-arabinofuranosyl-*β*-D-glucopyranoside	monoterpenoid glycosides	*S. officinal*	MeOH extract of the dried crushed roots	Su et al. (2018a)
113	(2*E*)-3,7-dimethyl-2,6-octadien-1-yl 6-O-*α*-L-arabinofuranosyl-*β*-D-glucopyranoside	monoterpenoid glycosides	*S. officinal*	MeOH extract of the dried crushed roots	Su et al. (2018b)
114	7-hydroxy-3,7-dimethyloctyl-6-O-*α*-L-arabinofuranosyl-*β*-D-glucopyranoside	monoterpenoid glycosides	*S. officinal*	methanol and aqueous extract of the roots	Su et al. (2018b)
115	(2*E*)-7-hydroxy-3,7-dimethyl-2-octenyl 6-O-*α*-L-arabinofuranosyl-*β*-D-glucopyranoside	monoterpenoid glycosides	*S. officinal*	methanol and aqueous extract of the roots	Su et al. (2018b)
116	(2*E*)-7-hydroxy-3,7-dimethyl-2-octenyl 6-O-*α*-L-arabinopyranosyl-*β*-D-glucopyranoside	monoterpenoid glycosides	*S. officinal*	methanol and aqueous extract of the roots	Su et al. (2018b)
117	(2*E*,6*Z*)-3,7-dimethyl-8-hydroxyoctadien-1-ol	monoterpenoid glycosides	*S. officinal*	70% EtOH extract of the air-dried and powdered	Guo et al. (2019)
118	8-hydroxygeraniol-1-O-(6-O-galloyl)-*β*-D-glucopyranoside	monoterpenoid glycosides	*S. officinal*	70% EtOH extract of the air-dried and powdered	Guo et al. (2019)
119	8-hydroxygeraniol-1-O-*α*-l-arabinofuranosyl-(1→6)-*β*-D-glucopyranoside	monoterpenoid glycosides	*S. officinal*	70% EtOH extract of the air-dried and powdered	Guo et al. (2019)
120	ethyl isobutyrate hexanal	essential oils	*S. albanica*	stems, leaves, and flowers in Thymol	Sabbatini et al. (2019)
121	*(Z)*-4-heptenalb octanal	essential oils	*S. albanica*	stems, leaves, and flowers in Thymol	Sabbatini et al. (2019)
122	*(Z)*-3-hexenol linalool oxide	essential oils	*S. albanica*	stems, leaves, and flowers in Thymol	Sabbatini et al. (2019)
123	*(Z)*-4-heptenal acetic acidc	essential oils	*S. albanica*	stems, leaves, and flowers in Thymol	Sabbatini et al. (2019)
124	*(Z)*-3-hexenol nonanal	essential oils	*S. albanica*	stems, leaves, and flowers in Thymol	Sabbatini et al. (2019)
125	linalool	essential oils	*S. albanica*	stems, leaves, and flowers in Thymol	Sabbatini et al. (2019)
126	*(E,Z)*-2,6-nonadienal *α*-terpineol	essential oils	*S. albanica*	stems, leaves, and flowers in Thymol	Sabbatini et al. (2019)
127	verbenone	terpenoids	*S. albanica*	stems, leaves, and flowers in Thymol	Sabbatini et al. (2019)
128	*(E)*-2-nonenal linalool	essential oils	*S. albanica*	stems, leaves, and flowers in Thymol	Sabbatini et al. (2019)
129	ethyl isobutyrate *(Z)*-3-hexenalb	essential oils	*S. albanica*	stems, leaves, and flowers in Thymol	Sabbatini et al. (2019)
130	*(E,Z)*-2,6-nonadienal ethyl isobutyrate *(Z)*-4-heptenalby	essential oils	*S. albanica*	stems, leaves, and flowers in Thymol	Sabbatini et al. (2019)
131	*(E,Z)*-2,6-nonadienal	essential oils	*S. albanica*	stems, leaves, and flowers in Thymol	Sabbatini et al. (2019)
132	octanal rose oxide	essential oils	*S. albanica*	stems, leaves, and flowers in Thymol	Sabbatini et al. (2019)
133	*(E,Z)*-2,6-nonadienal	essential oils	*S. albanica*	stems, leaves, and flowers in Thymol	Sabbatini et al. (2019)
134	acetic ancid	essential oils	*S. albanica*	stems, leaves, and flowers in Thymol	Sabbatini et al. (2019)
135	*α*-linolenic acid	fatty acid	*S. minor*	80% methanol extract of leaves, stems and roots	Sabbatini et al. (2019)
136	palmitic	fatty acid	*S. minor*	80% methanol extract of leaves, stems and roots	Karkanis et al. (2019)
137	linoleic acid	fatty acid	*S. minor*	80% methanol extract of leaves, stems and roots	Karkanis et al. (2019)
138	stearic	fatty acid	*S. minor*	80% methanol extract of leaves, stems and roots	Karkanis et al. (2019)
139	tricosylic	fatty acid	*S. minor*	80% methanol extract of leaves, stems and roots	Karkanis et al. (2019)
140	lauric	fatty acid	*S. minor*	80% methanol extract of leaves, stems and roots	Karkanis et al. (2019)
141	eicosatrie-noic acid	fatty acid	*S. minor*	80% methanol extract of leaves, stems and roots	Karkanis et al. (2019)
142	dihomo-*γ*-linolenic	fatty acid	*S. minor*	80% methanol extract of leaves, stems and roots	Karkanis et al. (2019)
143	behenic acids	fatty acid	*S. minor*	80% methanol extract of leaves, stems and roots	Karkanis et al. (2019)
144	2-phenylethylamine	amine	*S. officinal*	MeOH/H_2_O and EtOH/H_2_O extract of fresh flowers	Bunse et al. (2020)
145	*β*-L-arabinofuranoside	glycosides	*S. officinal*	95% EtOH extract of the dried roots	Wang et al. (2020b)
146	n-butyl-*β*-D-fructofuranoside	glycosides	*S. officinal*	95% EtOH extract of the dried roots	Wang et al. (2020b)
147	lyoniside	lignan	*S. officinal*	95% EtOH extract of the dried roots	Wang et al. (2020b)

n.d, no data.

## Pharmacological Effects Exhibited by Genus *Sanguisorba*

Many researchers have reported a variety of pharmacological effects of genus *Sanguisorba*, not only *in vitro,* but also a large amount of *in vivo* experimental data, involving anti-inflammatory, anti-cancer, anti-lipid peroxidation, anti-bacteria, anti-diabetes, hepatoprotective, and anti-obesity effects.

### Anti-Inflammatory Effects

*S. officinalis* has been used for the treatment of inflammatory diseases, including the airway inflammation in bronchial asthma (Lee et al., 2010), contact dermatitis (Jo et al., 2015), specific dermatitis (Park et al., 2015; Yang et al., 2016a), nephritis (Zhao et al., 2019), colitis (Shao et al., 2017; Fang et al., 2018; Yasueda et al., 2020), etc., for a long time.

Yu et al. (2011) reported that the ethanol extract of *S. officinalis* plants could block the production of representative inflammatory mediators nitric oxide (NO) and prostaglandin E2 (PEG2) at the transcription level in an *in vitro* model of RAW264.7 cells stimulated by 1 μg/ml lipopolysaccharide (LPS). In 2015, Yang et al. reported that the ethanol extract of *S. officinalis* plants could inhibit the production of pro-inflammatory chemokines in human keratinocytes (HaCaT) cells induced by tumor necrosis factor (TNF)-α/interferon (IFN)-γ; these cytokines are signal peptides involved in several inflammatory skin diseases. In the following year, the authors reported that the water extract of these plants exhibited the same anti-inflammatory effect in bone marrow-derived mast cells and HaCaT cells, demonstrating that the degranulation of immuno-globulin E (IgE)/antigen (Ag)-activated mast cells, phosphorylation of p38, and JNK in HaCaT cells were inhibited (Yang et al., 2016b). Seo et al. further confirmed the anti-inflammatory effect of the water extract of *S. officinalis* (HSO) in an *in vivo* mouse model induced by LPS (3 mg/kg), demonstrating that the oral consumption of HSO (5 or 25 mg/kg⋅day) significantly reduced the levels of serum as well as intraperitoneal interleukin 1β (IL-1β) in a dose-dependent manner along with improving the survival rate (Seo et al., 2018). Moreover, *S. officinalis* at a dose of 1 mg/ml reportedly activated autophagic activity and significantly inhibited 2% dextran sodium sulfate (DSS)-induced colitis, without damaging the liver, heart, and kidneys in mice (Yasueda et al., 2020).

The chemical components mainly responsible for the anti-inflammatory activity exhibited by root parts are phenolic compounds and linear monoterpenes (Su et al., 2018a). *In vitro* anti-inflammatory tests conducted on zebrafish indicate that the gallic acid group could be the key bioactive group in the terpene glycosides present in *S. officinalis*, and might function as regulators of the distribution of zebrafish macrophages (Guo et al., 2019). Polysaccharides from *S. officinalis,* when used at concentrations of 25 mg/ml and 100 mg/ml, exhibit evident antagonistic effects on P-selectin-mediated leukocyte adhesion, which is a promising target for the treatment of inflammation-related diseases (Tong et al., 2015). Two acidic polysaccharides purified from *S. officinalis* (Zhao et al., 2019), and ellagic acid (Seo et al., 2016), which is considered a marker component of *S. officinalis*, have demonstrated significant inhibition of the production of pro-inflammatory cytokines TNF-α and IL-6 in RAW264.7 cells stimulated by LPS *in vitro*. In addition, the acidic polysaccharides could effectively improve LPS-induced renal injury in mice by demonstrating acute anti-inflammatory activity (Zhao et al., 2019). Furthermore, ZYM-201, a methyl ester of triterpenoid glycoside, may ameliorate inflammation by inhibiting nuclear factor kappa-B (NF-κB) activation and downregulating the expression of costimulatory molecules on the surface of B cells stimulated by LPS (1 μg/ml).

### Antitumor Effects

Despite huge advances in various antitumor therapies, such as targeted therapy and immunotherapy, chemotherapy continues to be the most commonly used one for the treatment of tumors (Hemminki et al., 2020). Several extracts from genus *Sanguisorba* have demonstrated significantly greater toxic effects on a variety of tumor cells, compared to the non-tumor cells, *in vitro*.

It is reported that the water extract of *S. officinalis,* when used at a relatively low dose (IC_50_ < 200 μg/ml) and in combination with 5-fluorouracil, could increase the cytotoxic effect on two colorectal cancer cell lines HCT-116 and RKO by promoting the reactive oxygen species-mediated mitochondrial caspase-dependent apoptotic pathway (Liu et al., 2016). A similar effect was observed when ellagic acid isolated from the alcohol extracts of *S. officinalis* was used in combination with cisplatin treatment (Tan et al., 2019), as reported by numerous scientific studies (García-Niño and Zazueta, 2015; Ceci et al., 2018).

Methanol extracts of *S. officinalis* (40, 80, or 120 μg/ml) have demonstrated significant cytotoxic activity against human prostate cancer cells *via* an intrinsic apoptotic pathway (Choi et al., 2012a), in addition to inhibiting the proliferation of human breast cancer cell lines MCF-7 and MDA-MB-231 by inducing S-phase arrest and triggering the mitochondrial pathway of apoptosis (Wang et al., 2012), and causing the blockage of the G1 phase in B16F10 melanoma cells (Tan et al., 2019).

Triterpenes isolated from *S. officinalis* roots play a major role in the antitumor effect exhibited by this species and demonstrate significant cytotoxicity in various human tumor cell lines, such as BGC-823 cells (human gastric cancer), HeLa cells (human cervical cancer), MCF-7 cells (human breast cancer), SGC-7901 cells (human gastric adenocarcinoma), A549 (human lung cancer) and NCI-H460 cells (human large cell lung cancer), and SK-Hep1 (hepatoma cell) and HepG2 cells (human hepatocellular carcinoma), *in vitro* (Hu et al., 2015; Wang et al., 2019a). Interestingly, Mazzio and Soliman (2017) reported that the main role of triterpenes in HeLa cells is to resist mitosis rather than causing cytotoxicity.

Ziyuglycoside I (ZY-I) from *S. officinalis* roots is reported to induce mitochondria-dependent apoptosis in human retinoblastoma WERI-RB-1 cells, which are representative of the most common intracellular malignancy, by activating P53 in a concentration-dependent manner (Zhu et al., 2017). According to an *in vivo* experiment, 3,3′,4′-trimethylellagic acid (TMEA, an ellagic acid) derived from *S. officinalis* roots exhibited dose-dependent downregulation of the expression of anti-apoptotic factors CD31 and Bcl-2 and upregulation of the expressions of apoptotic factors Bax and caspase-3 in the allograft tumor of SW620 nude mice (Bai et al., 2020b).

Zhu et al. (2013a) were the first to discover that ziyuglycoside II (ZY-II) from *S. officinalis* roots could inhibit the growth of two classic human breast cancer cell lines MCF-7 and MDA-MB-231 and induce the apoptosis of human colon cancer cells HCT116 and SW480 (Lkhagvasuren and Kim, 2019). The inhibition of proliferation of hepatocellular carcinoma cells caused by ZY- II is reported to be mainly due to increased apoptosis, accumulation of reactive oxygen species, and cell cycle arrest in the G0/G1 phase (Liao et al., 2020), although the apoptosis of gastric carcinoma cells BGC-823 induced by ZY- II applied at a concentration of 25 µM would not induce cell cycle arrest (Zhu et al., 2013b). Oral intake of ZY- II (1 or 5 mg/kg) three times per week could reportedly reduce the nuclear factor kappa-B-positive cells and the levels of inflammation-related proteins, promoting azoxymethane-induced colon cancer in BALB/c mice (Cheon and Kim, 2019).

In addition to targeting the intrinsic pathway of apoptosis, inhibiting the formation of blood vessels that supply oxygen and essential nutrients to cancer cells is considered another promising approach to cancer treatment.

ZY- II could reportedly inhibit the proliferation, migration and tubule formation of human umbilical vein endothelial cells (HUVECs), probably by blocking the signaling pathway mediated by the vascular endothelial growth factor receptor 2(VEGFR2) and fibroblast growth factor receptor 1 (Nam et al., 2017). TMEA could inhibit the growth of breast cancer cells and angiogenesis of human umbilical vein endothelial cells (Wang et al., 2012). In addition, TMEA could combine with VEGFR2 in the functional area to inhibit the proliferation, migration, tube formation and VEGF expression and downstream signals in HUVECs (Bai et al., 2020a).

The extract of *S. minor* (Cuccioloni et al., 2012) appears to be as effective as that of *S. officinalis* (Li et al., 2019) in restraining the plasmin-mediated migration of cancer cells and demonstrating excellent antitumor ability against certain cancer cell lines, such as HepG2 (Vanzani et al., 2011; Karkanis et al., 2019). It is noteworthy that compared to the leaf and stem extracts of *S. officinalis,* its root extracts exhibit a stronger anticancer activity against most cancer cell lines. Whether this difference is related to the higher content of phenolic compounds in the root system requires further investigation. Although it is reported that under different planting conditions, the roots of half-rate fertilizer (330 kg/ha) having the highest content of total phenolic compounds indeed exhibit an increased cytotoxic effect on tumor cell lines (Finimundy et al., 2020). Despite *S. minor* being a common part of the human diet in the Mediterranean region, its complete potential has not been explored so far as the tissues of this plant might serve as a potential source of natural bioactive compounds that could further be used in medicine.

Currently, while the antitumor activity exhibited by the plants of genus *Sanguisorba* has been verified in a variety of tumor cell lines *in vitro*, the *in vivo* experiments remain insufficient to validate these effects.

### Hemostatic Effects

In China, South Korea, Japan, Siberia, and Europe, *S. officinalis* is frequently used as a hemostatic agent. In China, *S. officinalis* plants are often transformed into charcoal of *S. officinalis* and used clinically to control bleeding. An experimental study on the effect of raw *S. officinalis* and *charred sanguisorba* based on the tail-breaking and capillary method demonstrated that while both forms could significantly shorten the duration of bleeding and reduce the clotting time in mice, the effect of charred *sanguisorba* was significantly stronger than that of raw *S. officinalis* at an equivalent dose (Zhou, 2014). According to the research of Ma et al. (2017), thermal analysis techniques could be used to precisely control the temperature and thereby determine the energy changes occurring during the partial carbonizing process of *S. officinalis*.

Consistent with the traditional usage of the plants of genus *Sanguisorba*, a large number of studies have reported the hemostatic effects exhibited by the plants of this genus *in vivo* as well as *in vitro*. Based on the evidence provided by both the Szejk et al. (2017b) and Liu et al. (2018c), the polysaccharide-polyphenolic conjugates in Rosaceae/Asteraceae plants exhibit various biological activities, such as anticoagulation, radiation protection, anti-platelet and bronchodilatory effects. The polyphenol-polysaccharide conjugate in dried and flowering parts of *S. officinalis*, the anticoagulant activity of which is reportedly mediated mainly by heparin cofactor II (Pawlaczyk-Graja et al., 2016), are capable of selectively protecting the normal lymphocytes from radiation damage (Zbikowska et al., 2016; Szejk et al., 2017a; Szejk-Arendt et al., 2019).

The hemostatic activity of seven compounds isolated from *S. officinalis* was evaluated using the goat anti-human α2-plasmin inhibitor kit and purified α2-plasmin inhibitor-specific antibody. The results demonstrated that terpene glycosides were responsible for the hemostatic activity, with ZY-I as the main hemostatic component that demonstrated the strongest hemostatic activity (88.7%) at a concentration of 0.094 mg/μL (Sun et al., 2012). It was also reported that ZY- I does not exhibit a strong tissue factor -inhibitory activity, and the chemical modification (degumming, esterification, etc.) of its structure, ZY-I deglycoside methyl ester (IC_50_ = 0.46 mM) improves its inhibitory activity against TF and TNF-α (Jae et al., 2006).

### Antioxidant Activity

Polysaccharides are the active ingredients of several traditional medicines (Liu et al., 2018a) and the natural antioxidant ingredients of various potential phytopharmaceutical resources (Dkhil et al., 2016), with a long history in ethnopharmacology and little edible toxicity.

*S. officinalis* is regarded as a herbal medicine with extremely strong antioxidant properties (Liao et al., 2008), which are often evaluated using the DPPH (2,2-dipheny1-1-picrylhydrazy1) removal method and the yeast oxidative stress of the *S. officinalis* polysaccharide. Polysaccharides, when used in a dose range of 552–977 μM, exhibit strong radical-scavenging activity and relieve the *Saccharomyces cerevisiae*-caused oxidative stress induced by oxidants in the body (Zhang et al., 2012a). Ravipati et al. (2012) believed that the antioxidant and anti-inflammatory activities of *S. officinalis* are significantly associated with its phenolic, flavonoid and trace metal contents. In the same year, four phenolic compounds were identified and isolated from the roots of *S. officinalis*, among which fisetinidol-(4*α*-8)-catechin exhibited the strongest antioxidant activity (Zhang et al., 2012b). *S. officinalis* was also reported to prevent ischemic brain injury in cultured rat model of cortical neurons and middle cerebral artery occlusion and was proposed as a promising drug for the treatment of neurodegenerative diseases, such as stroke and Alzheimer’s disease (Nguyen et al., 2008).

Different methods used for obtaining the extract of *S. officinalis* could result in different contents of total phenols, flavonoids, and terpenoids, and consequently, in different antioxidant activities demonstrated *in vitro*. When the extracts of *S. officinalis* roots were obtained using cold water (CWE), hot water (HWE) and methanol (ME), the obtained ethyl acetate fractions exhibited dose-dependent free radical scavenging ability (SC) values, as follows: the best SC_50_ value of 7.58 µg/ml is obtained for HWE, followed by CWE (12.14 µg/ml), ME (16.74 µg/ml), CWE-EA (19.14 μg/ml), HWE-EA (35.81 μg/ml), and ME-EA (52.46 μg/ml) (Kim et al., 2018a). The chemical compositions of the *S. officinalis* extracts obtained using different methods are also different. The methanol extract presents a flavonoid content that is approximately three times higher than that of the water extract, while the total phenolic content in the water extract is relatively higher (Gawron-Gzella et al., 2016). Moreover, the total phenol content in the methanol extract is two times lower than that in the acetone extract obtained from the same plant (Ginovyan et al., 2020).

The phytochemicals present in *S. officinalis* are a potent source of exogenous antioxidants that could scavenge the free radicals inside the body, thereby diminishing the effects of photoaging (Yokozawa and Chen, 2001; Mukherjee et al., 2011). Several *in vivo* and *in vitro* studies have demonstrated that ZY-I increases the contents of collagen and elastic fibers in a dose-dependent manner and also inhibits the production of the collagen-degrading enzyme MMP-3 in the skin, thereby demonstrating an anti-wrinkle effect (Kim et al., 2008; Yun et al., 2019). In a randomized double-blind placebo experiment conducted with 21 Japanese women, it was observed that at the cellular level, it was the *S. officinalis* root extract, rather than ziyuglycoside-I, that inhibited the hyaluronic acid degradation and consequently exerted the anti-wrinkle effect (Yoshida et al., 2018).

Interestingly, when the chemical constituents of the root, stem, and leaf extracts of *S. minor* were analyzed, it was revealed that the content of polyphenols was significantly higher in the stem and leaf extracts compared to that in the root extracts. Moreover, the highest value of total polyphenol content in *S. minor* was 258 mg/100 g, and the content of polyphenols was particularly high (98.2 mmol total phenol/kg) (Romojaro et al., 2013b; Karkanis et al., 2014). Such high contents of biologically active constituent compounds and a strong antioxidant activity are responsible for the role of *S. minor* in the inflammatory process caused by excessive free radical oxidation, such as the inflammatory process associated with Alzheimer’s disease (Ceccanti et al., 2019). In addition, the dry powder of *S. minor* may be utilized for concentrating the vegetable oils with a low natural antioxidant content, such as sunflower oil and corn oil, which would enhance the overall oxidative stability of these oils (Finimundy et al., 2020).

Therefore, due to its rich ingredients and various biological functions, *S. minor* could be used as a high-in-antioxidant functional food for nutritional supplementation and a natural antioxidant that would replace the artificially synthesized ones, thereby improving the diversity of ingredients in modern cooking. It may also be formulated as a drug to prevent or treat diseases caused due to oxidative stress.

### Antibacterial Effects

Antibiotics represent an important class of therapeutic agents used for the treatment of bacterial infectious diseases (Sun et al., 2004). Unnecessary and excessive use of antibiotics is particularly concerning as this could lead to several adverse drug events, including allergic reactions, end-organ toxic effects, subsequent infection with antibiotic-resistant organisms, and *Clostridium difficile* infections (Tamma et al., 2017). The demand for novel antibacterial drugs capable of effectively combating drug-resistant microorganisms has increased to a great extent (Wright, 2017) and plant materials are generally preferred now for use as natural antibacterial agents in the treatment of various infections (Ginovyan et al., 2020).

Methicillin-resistant *Staphylococcus aureus* (MRSA) is an important nosocomial pathogen that is resistance to many antibiotics and is, therefore, associated with serious infections. Ethanol extracts of *S. officinalis* (50 mg/ml) are reported to play important roles in the inhibition of MRSA. At high concentrations (>7.5 mg/ml), *S. officinalis* remarkably inhibited the growth of MRSA. However, at low concentrations (<2.5 mg/ml), *S. officinalis* could only cause a slight inhibition of the growth of MRSA (Chen et al., 2015).

The ethanol and methanol extracts of the underground parts and rhizomes of *S. officinalis* exhibit significant antibacterial activity against Gram-positive bacteria, Gram-negative bacteria, and fungi, with all herbal extracts demonstrating a minimum inhibitory concentration value of 0.07–2.50 mg/ml (Gawron-Gzella et al., 2016). Moreover, *S. officinalis* might also have the potential to treat local acne owing to the anti-propionic acid activity of the different extracts of this species (Kim et al., 2018b). The antimicrobial potential of the crude extracts of *S. officinalis* against various bacterial and yeast strains has been demonstrated using the TLC-Bioautographic Technique (Ginovyan et al., 2020). Furthermore, a purified mulberry polyphenol extract exhibited strong antibacterial activity against *Bacillus subtilis*.

The research team of Karkanis et al. evaluate the antibacterial property of *S. minor* under different growth conditions which could be related to the content of phenolic compounds and the composition of different phenolic compounds. The extracts from the roots demonstrate a higher antibacterial ability compared with the aerial parts of plants are probably due to the higher content of phenolic compounds in the roots. *S. minor* extracts were tested for the antibacterial activity of six strains of *Bacillus cereus*, *Enterobacter cloacae*, *Escherichia coli*, *Listeria monocytogenes*, *Staphylococcus aureus* and *Salmonella typhimurium*. The MIC and MBC value extracts ranging from 0.075 to 0.45 mg/ml and 0.15–0.60 mg/ml, respectively. At the same time, *S. minor* extract also showed antifungal activity.

### Antiviral Effects

Hepatitis B virus (HBV) causes acute and chronic liver disease, both of which place a serious burden on global health due to the associated morbidity and mortality (Indolfi et al., 2019). The limitations of conventional antiviral drugs, such as concerns associated with long-term usage, drug resistance, and virological relapse, have rendered the infectious diseases caused by HBB almost incurable so far. KCT-01, a novel herbal formula developed for working against the HBV, is composed of mugwort, *S. officinalis* and turmeric. KCT-01, when applied at 250 μg/ml, was sufficient to reduce the secretion of both HBsAg and HBeAg in HepG2 cells to below 50% compared to the mock-treated control. The antiviral effect of KCT-01 was confirmed in a mouse hydrodynamic injection model, which demonstrated inhibition of HBV replication and the production of inflammatory cytokines, while no toxicity was observed, indicating that KCT-01 alone or in combination with entecavir has the potential to serve as an antiviral agent (Kim et al., 2018a).

Previously, a study had demonstrated that the levels of extracellular HBV virion DNA were decreased, and the secretion of HBsAg was inhibited in a dose-dependent manner with the use of *S. officinalis* extract (SOE) at concentrations ranging from 64 to 128 μg/ml (Kim et al., 2001). In addition, the extract exhibited significant inhibitory effects on both CCR5 and CXCR4 tropic human immunodeficiency virus-1 (ADA and HXB2), with IC_50_ values of 1.91 ± 0.16 μg/ml and 3.70 ± 0.53 μg/ml, respectively. SOE also inhibited simian immunodeficiency virus infection, although it failed to block the vesicular stomatitis virus (VSV), SARS-CoV, and influenza H5N1 pseudoviruses (Liang et al., 2013). The methanol extract of *Sanguisorba officinalis*; however, exerts a certain inhibitory effect on the replication of coronavirus (Gawron-Gzella et al., 2016). Activity high-throughput screening assay was employed to screen 190 herbal extracts for the evaluation of their biological activities, and it was revealed that 14 of these extracts, including the extract of *S. officinalis*, significantly inhibited the activity of neuraminidase (the main drug target for anti-influenza virus therapy), with IC_50_ values of these extracts ranging from 4.1 to 9.6 μg/ml (Liu et al., 2018b). ZY-II reportedly inhibits the cell growth and rotavirus replication in a dose-dependent and time-dependent manner, in addition to inhibiting the TLR4/NF-κB pathway and the inflammatory response, while improving rotavirus-induced diarrhea (Liao et al., 2020).

The majority of the plants belonging to genus *Sanguisorba* exhibit a certain level of antiviral activity, particularly the extract of *S. minor*, which was demonstrated to significantly inhibit herpes simplex virus type 1 (DNA virus) and VSV (RNA virus) at non-toxic concentrations of 50–125 mg/ml (Choi et al., 2012b).

Further detailed and comprehensive research should be planned and executed to explore and develop improved drugs capable of controlling the human immunodeficiency virus, HBV, and other viruses.

### Neuroprotective Effects

Natural compounds derived from medicinal and edible plants have attracted the attention of scholars exploring novel treatment methods for neurological diseases. Catechin (and not gallic acid) present in the root of *S. officinalis* in the concentration range of 10–50 μg/ml reportedly inhibits the neuronal death induced by H_2_O_2_ (100 μM) by eliminating the free radical activity in neurons. In a study conducted using the *in vivo* model of ischemic brain injury in rats with middle cerebral artery occlusion, the oral administration of 10 or 30 mg/kg *S. officinalis* was observed to confer a significant protective effect in terms of the volume of cerebral infarction and cerebral edema in rats, which was the first proof of Neuroprotective effect in anti-oxidant-caused brain damage (Nguyen et al., 2008). In the same year, another study reported that the methanol extract of *S. officinalis* could prevent Aβ(25-35)-induced neuronal cell damage *in vitro* and that the gallic acid isolated from this extract conferred a certain level of protection against the neurotoxic effect to the Aβ(25-35)-induced cortical neurons in rats (Ban et al., 2008).

The amyloid hypothesis has dominated the research on Alzheimer’s disease (AD) for nearly 30 years now (Ennerfelt and Lukens, 2020). This implies that *S. officinalis* could provide a novel potential therapeutic approach for controlling the progress of neurodegeneration in an AD-affected brain, while also being a promising drug for the treatment of other neurodegenerative diseases, such as stroke.

Sanguiin H-11 (SH-11) derived from the root of *S. officinalis* also exhibits a strong antioxidant activity. SH-11 acts as a powerful antioxidant that significantly reduces the glutamate-induced accumulation of reactive oxygen species and a calcium ion influx in mouse clonal hippocampal HT22 cells. Apoptotic cells exhibit effective neuroprotective activity *via* glutamate-induced phosphorylation of mitogen-activated protein kinases, including the extracellular signal-related kinases 1/2, c-Jun N-terminal kinase, and p38, and this activity was decreased significantly by SH-11 (Song et al., 2019).

### Hematopoietic Effects

Clinical practice in China in the past few decades has confirmed that the extract of *S. officinalis* increases the number of white blood cells and reduces the bone marrow toxicity caused by antitumor treatments.

Myelosuppressive mice induced by exposure to cyclophosphamide and ^60^Co-γ radiation for 13 days were tested for the efficacy of total saponins of *S. officinalis* at the doses of 1.6 mg/kg, 0.8 mg/kg and 0.4 mg/kg administered orally. The results demonstrated that survival was promoted through the activation of focal adhesion kinase (FAK) and extracellular signal-regulated kinase 1/2 (Erk1/2) and the modulation of cytokine production in the bone marrow (Chen et al., 2017) Two ellagic acid compounds isolated from the ethyl acetate extracts of *S. officinalis* root promoted megakaryocyte progenitor cells in a dose-dependent (10 μg/ml or 20 μg/ml) and time-dependent (4, 8 and 12 days) manner, leading to their proliferation and induction of megakaryocyte differentiation (Gao et al., 2014).

### Hypoglycemic and Lipid-Lowering Effects

Obesity, hyperglycemia, and hypertension or dyslipidemia are the three medical conditions that usually occur simultaneously in patients and are often described as metabolic syndrome that increases the risk of diabetes and cardiovascular disease in the affected patients (Samson and Garber, 2014). In China, *Sanguisorba* × *tenuifolia* Fisch. ex Link (*S. tenuifolia*) is used commonly for treating diabetes. The ethyl acetate layer in the alcohol extract of the roots of *S. tenuifolia* was observed to be rich in triterpenes, which could inhibit plasma glucose levels in diabetic rats induced by alloxan. These triterpenoids have been reported for the first time in this plant variety, and they demonstrate inhibitory activity against α-glucosidase (Kuang et al., 2011). These plants are used as an alternative medicine to replace *S. officinalis* in diabetes.

In comparison to ZY-I, chemically modified ziyuglycoside II methyl ester (ZG02-ME) exhibits a better performance in the treatment of type 2 diabetes. A single dose of ZY- I or ZG02-ME (5 mg/kg body weight) each day for 1 week is capable of lowering the blood sugar levels by 2.6% or 11.4%, respectively, in addition to significantly decreasing the levels of glycated hemoglobin (HbA1c) and serum insulin. Further evaluation of the anti-diabetic effect of ZG02-ME consumed for 4 consecutive weeks revealed that it could significantly reduce blood glucose levels in a dose-dependent manner, by 11.1, 17.6 and 22.4% at the dose of 1, 3 and 5 mg/kg, respectively (Son et al., 2015). Oral administration of ZYM-201 sodium succinate (1–10 mg/kg), produced as a chemical modification of the triterpene glycosides isolated from *S. officinalis*, reduces the diet-induced body weight and liver weight, and returns the serum triglyceride and total cholesterol levels to their normal ranges in hyperlipidemic rats (Choi et al., 2011) as well as in Hyperlipidemic rats with hyperglycemia (Choi et al., 2012a). In addition, this compound normalizes the changes that had occurred in the lipid metabolism due to hyperglycemia and high-fat diets, allowing its use for improving alcohol-induced hyperlipidemia (Cho et al., 2014).

## Clinical Investigations on *S. officinalis*

The clinical incidence of malignant tumors has been increasing in recent years. Radiotherapy is one of the main methods used for treating malignant tumors. However, radiotherapy often causes bone marrow suppression, which greatly reduces the peripheral white blood cell count greatly and affects the outcomes of radiotherapy to a certain extent. Drugs such as Squalanol are used commonly in the treatment of leukopenia after radiotherapy, although these drugs are more likely to cause adverse reactions in patients after treatment, which reduces the treatment tolerance.

*Sanguisorba officinalis* white tablet has been clinically used for the treatment of leukopenia caused by continuous application of radiotherapy and chemotherapy in cancer for over 10 years (Zhu et al., 2020b). This method of tablet treatment effectively compensated for the deficiency of shark liver alcohol and was widely accepted by patients. Ziyuglycoside I, which is one of the main active ingredients in the *Sanguisorba officinalis* white tablet, is clinically proven to reduce leukopenia. Since ZY-I exhibits low solubility and permeability when administered orally, a ZY-I-loaded self-microemulsifying drug delivery system has been developed to improve the bioavailability and intestinal absorption of ZY-I, thereby increasing the pharmacokinetics and pharmacodynamic activity of leukocyte (Xiong et al., 2019). In addition, through searching Cochrane Library database (Cochrane Library, 2021), records in the four trials, there prescription containing composition *S. officinalis* treatment of ulcerative colitis (Table 2).

**TABLE 2:** Summary of the contemporary clinical uses of *S. officinalis*.

No.	Type and number of participants	Herbal constituents	Herbal drug effects	References
1	1822 patients with bleeding haemorrhoids	Radix *Sanguisorbae* formulations	stop bleeding from haemorrhoids	Gan et al. (2010)
	(age range,17–87 years old)			
2	21 healthy Japanese women	*S. officinalis* root extract and ziyuglycoside I	anti-wrinkle activity on human facial skin	Yoshida et al. (2018)
	(age range, 34–56 years old)			
3	20 females in good general health	ziyuglycoside I	anti-wrinkle activity	Kim et al. (2008)
	(age range, 35–53 years old)			
4	120 confirmed diagnosis of steroid-dependent ulcerative colitis	Radix *Sanguisorbae* formulations	steroid-dependent ulcerative colitis	Zheng et al. (2017)
	(age range, 18–65 years old)			
5	60 patients with mild-to-moderately initial onset or relapsed active ulcerative colitis (UC)	Enema of Guanchang Recipe (6 herbs including Radix *Sanguisorbae*)	treat active UC	He et al. (2012)
	(age range, 28–52 years old)			

Data from large-scale, randomized, double-blind, multi-center trials are needed to confirm the efficacy and safety of the clinical use of traditional Chinese medicine. These clinical trials should use standardized efficacy indicators, and should include an assessment of adverse events.

## Toxicity

So far, there have been limited reports on the toxicity caused by the different species of *Sanguisorba* in humans, and the major safety concern related to these species have been confined to the veterinary field. To date, no harmful components, such as alkaloids, have been reported in *S. minor* and *S. officinalis* (Sabbatini et al., 2019). Egorova et al. (2018) investigated the medicinal products of *S. officinalis* (rhizome and root) for the presence of heavy metals and other ecotoxic substances and reported no toxicity, thereby providing evidence for the safety and non-toxicity of *S. officinalis*. Nonetheless, to establish the safety of plants belonging to genus *Sanguisorba*, further research involving a comprehensive safety assessment is necessary.

## Perspectives and Discussion

**FIGURE 5:**
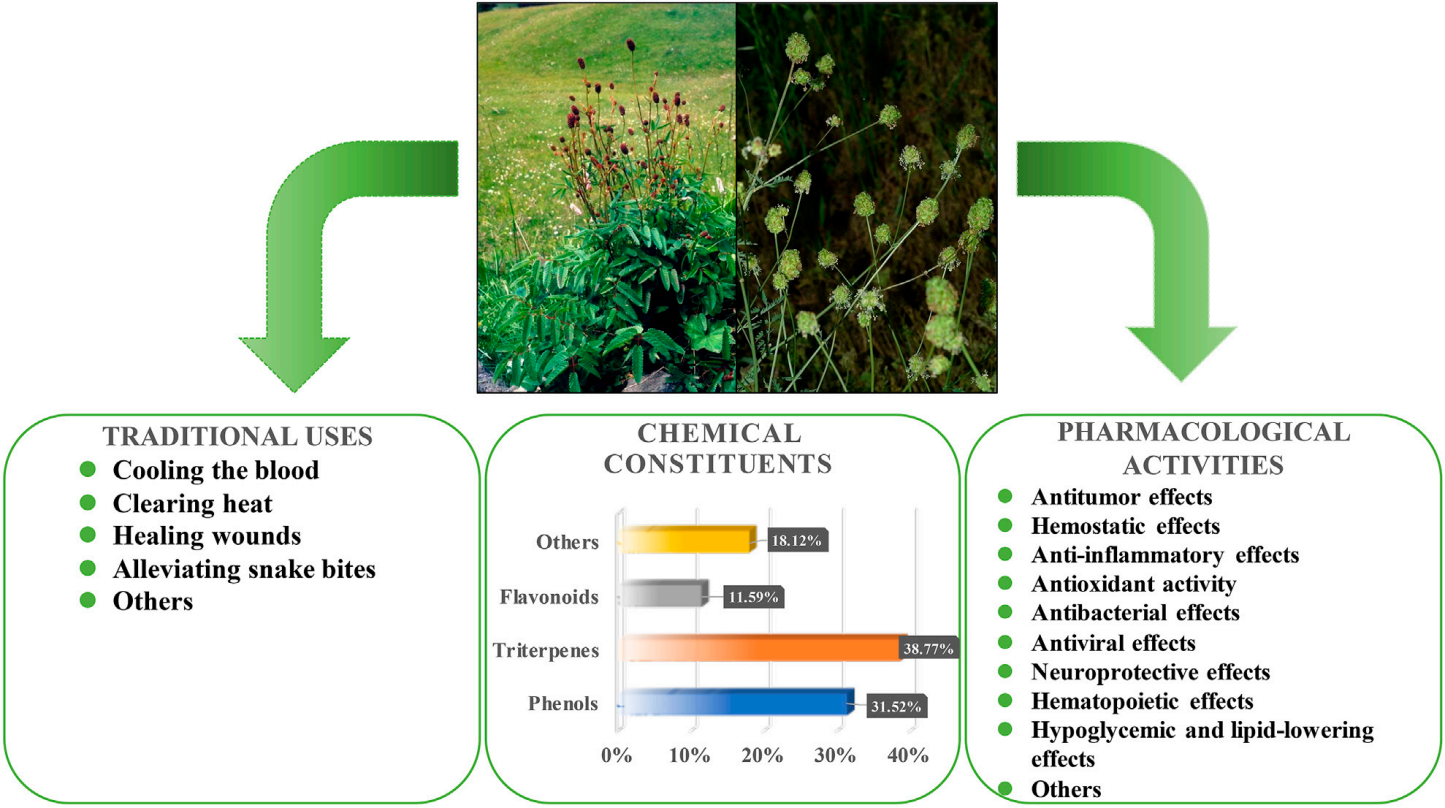
Traditional applications, chemical compositions and pharmacological activities of *S. officinalis* and *S. minor.*

This review uses *S. officinalis* and *S. minor* as representatives of the genus *Sanguisorba*, providing a comprehensive understanding of the traditional application, chemical composition and pharmacological activities of genus *Sanguisorba* (Figure 5). It is well recognized that the various ingredients present in herbs, besides producing a synergistic beneficial effect, also reduce the toxicity caused by a single compound. However, the biggest problem encountered when using traditional herbal treatments is the inability to ensure the quality and consistency of the herbal extracts. In recent years, genus *Sanguisorba* has been attracting increasing attention, with several of its traditional uses explored and investigated for potential use in current medicine. However, the reports on the alkaloids or other harmful ingredients, toxicity to target organs, and the safety of *Sanguisorba* are scarce. Therefore, further studies on the toxicity and pharmacokinetics of the genus *Sanguisorba* plants are warranted.

With advancements in the separation and purification of the active ingredients in the plants of genus *Sanguisorba*, the detailed research of its species pharmacology and molecular mechanism has also improved greatly, which could, in turn, ensure further precise pharmacological effects. Additionally, the separation of the chemical components of genus *Sanguisorba* may become a good candidate for chemotaxonomic markers. The application of novel technologies, such as high-throughput screening, would also greatly improve the probability and quality of novel drug discovery and subsequent clinical implications in the future.
